# Clinical efficacy and prognostic indicators for lower limb pedalling exercise early after stroke: Study protocol for a pilot randomised controlled trial

**DOI:** 10.1186/1745-6215-12-68

**Published:** 2011-03-07

**Authors:** Nicola J Hancock, Lee Shepstone, Philip Rowe, Phyo Kyaw Myint, Valerie Pomeroy

**Affiliations:** 1 Institiute of Health and Social Sciences, Faculty of Health, University of East Anglia, Norwich, UK; 2 Department of Bioengineering, University of Strathclyde, Glasgow, UK

## Abstract

**Background:**

It is known that repetitive, skilled, functional movement is beneficial in driving functional reorganisation of the brain early after stroke. This study will investigate a) whether pedalling an upright, static exercise cycle, to provide such beneficial activity, will enhance recovery and b) which stroke survivors might be able to participate in pedalling.

**Methods/Design:**

Participants (n = 24) will be up to 30 days since stroke onset, with unilateral weakness and unable to walk without assistance. This study will use a modified exercise bicycle fitted with a UniCam crank. All participants will give informed consent, then undergo baseline measurements, and then attempt to pedal. Those able to pedal will be entered into a single-centre, observer-blinded randomised controlled trial (RCT). All participants will receive routine rehabilitation. The experimental group will, in addition, pedal daily for up to ten minutes, for up to ten working days.

Prognostic indicators, measured at baseline, will be: site of stroke lesion, trunk control, ability to ambulate, and severity of lower limb paresis.

The primary outcome for the RCT is ability to voluntarily contract paretic lower limb muscle, measured by the Motricity Index. Secondary outcomes include ability to ambulate and timing of onset and offset of activity in antagonist muscle groups during pedalling, measured by EMG.

**Discussion:**

This protocol is for a trial of a novel therapy intervention. Findings will establish whether there is sufficient evidence of benefit to justify proceeding with further research into clinical efficacy of upright pedalling exercise early after stroke. Information on potential prognostic indicators will suggest which stroke survivors could benefit from the intervention.

**Trial Registration:**

ISRCTN: ISRCTN45392701

## Background

### Therapy early after stroke

In the first few weeks after stroke, the brain is 'primed' for neurological recovery in response to rehabilitation training [1]. Indeed, Cramer [2] describes a 'golden period' for initiating restorative therapies, starting in the first days after onset and continuing for several weeks. However, animal studies on early therapy are equivocal. Kozlowski et al [3] demonstrated an increase in lesion size following early training and proposed a 'use-it-but-don't-overuse-it' strategy in this period. In contrast, Biernaskie et al [4] found that rats given enriched rehabilitation training from day five after an induced lesion demonstrated a marked improvement in recovery, whilst those given similar training beginning at day 30 improved no more than controls.

Whilst animal studies provide insights into brain changes underlying recovery, caution must be observed in generalising to human populations. Nonetheless, clinical studies do support early rehabilitation intervention to improve outcomes [5,6]. It is also possible that, if rehabilitation onset is delayed, patients might establish compensatory behaviours that could impact negatively on recovery of useful functional activity [7,8]. Additionally, National Clinical Guidelines for Stroke in the United Kingdom advise that people with acute stroke should be mobilised as early as possible [9]. However, the optimal dose and type of physical therapy required to drive useful functional reorganisation in early stroke survivors with different clinical characteristics remains unknown.

### Repetitive, functional training early after stroke

In the first days to weeks after onset, stroke survivors can present particular therapeutic challenges. Leg weakness is often substantial and the ability to contract paretic muscle sufficiently to be able to take part in functional, relevant activity, such as walking training, can be severely compromised. There are interventions which can be used to improve ambulatory capacity early after stroke-including treadmill training with and without partial body weight support, and walking facilitated 'hands-on' by therapists-but these are often time-consuming and likely to require extensive physical assistance from one or more members of a therapy team. The effort required from both patient and therapist(s) is often too great to enable more than a few repetitions of activity. This dose might be too low for effect.

Although the number of repetitions of an activity needed to facilitate human brain reorganisation has not been established, animal model studies suggest that 300-400 repetitions in a 30 minute session might be needed [10]. Repetition of motor activity has been demonstrated to produce changes in cortical representation maps [10,11], and may be an important consideration in rehabilitation programmes.

Repetition of motor activity alone, however, is not a sufficient driver to induce functional reorganisation of cortical networks. Motor skill acquisition, or motor learning, has been demonstrated to play a central role, in both animal [12] and human [13,14] studies. It has also been suggested that there may be benefit from goal-directed functional activity associated with normal afferent stimulation [15]. The salience of a task is an important consideration in rehabilitation programmes [7]. Indeed, current clinical guidelines suggest that functional, task specific activity is a key component of rehabilitation after stroke; gait re-training to improve independence in walking is such a functional activity and a principle goal for many patients [9]. Such evidence might suggest that optimal rehabilitation programmes should involve task specific activity and increasing levels of motor skill [16].

Therapists are therefore challenged to find strategies that enable repetitive, relevant and skilled activity in early stroke survivors. However, it remains unknown a) which specific physical therapies might drive brain reorganisation and motor recovery and b) which patients might respond best to which therapies.

### Cycling as a potential therapeutic activity early after stroke

Cycling is a functional activity that has potential to benefit patients when used as an adjunct to therapy after stroke [17]. It requires that agonist and antagonist muscles are contracted reciprocally and in a similar pattern to that required for walking [18]. Therefore, it is a repetitive muscle activity that may be beneficial in retraining gait [19]. Indeed, pedalling may facilitate phasic, co-ordinated muscle activity even in patients with severe hemiparesis [20]. Whilst familiar to many stroke survivors, reciprocal pedalling is likely to require re-acquisition of motor skill following the onset of hemiparesis.

Clinically, there is therefore potential to use static cycling for repetitive, co-ordinated exercise training as part of stroke rehabilitation programmes aiming to address deficits in motor function. However, the evidence in support of cycling interventions is preliminary. The early findings from our ongoing systematic review are that, whilst research into aerobic capacity after stroke has often incorporated a cycling paradigm [21-23], few trials have specifically evaluated the effects of cycling exercise on motor function early after stroke. There are some indications that cycling activity may have a positive effect on strength, reciprocal activation of antagonistic muscle groups and balance in stroke survivors in the sub-acute and chronic stages but cautious interpretation of these results is required for a number of reasons: sample sizes were relatively small (n = 24 [24]; n = 17 [20]; n = 8 [25]), exact time since stroke onset was not specified [24] and findings related to a single session which was not repeated over time [20].

In addition, much of this work has used a recumbent position with a standard leg cycle ergometer for cycling exercise [20,22,24,25]. Although suitable for cardiovascular training, this position does not replicate the upright posture needed for walking. We propose that cycling to provide functional training of the lower limbs early after stroke is best provided in an upright posture, in order to maximise potential for activity in major lower limb muscle groups, in a posture similar to walking. Indeed, muscle activation patterns during pedalling are not fixed and are modified according to body position [26,27] and heightened levels of activity in quadriceps and hamstrings have been demonstrated in more upright pedalling postures [26].

We have therefore adapted a standard exercise bike a) to provide trunk support in an upright pedalling posture and b) to maximise opportunities for patients with severe lower limb weakness to pedal, with use of the UniCam crank (UniCam Inc, Emerson, New Jersey, USA; see *instrumentation*). This crank enables a reduced circumference of the pedalling circle on the paretic side, where position 2 (P2) is the smallest circle and position 9 (P9) the largest (i.e normal pedalling). A preliminary study (unpublished, 2004/05, Wandsworth UK, Local Research Ethics Committee 03.0102) has demonstrated that stroke survivors can: a) pedal the modified exercise cycle for up to ten minutes with no adverse effects, and b) tolerate the different positions of the right and left pedals. Participants were included in this observational study if they were at least 14 days after stroke onset, able to sit without support for one minute, able to follow a one-stage command, previously independently mobile but now unable to mobilise and having no other limiting disease process or pathology.

### Potential prognostic indicators for therapeutic interventions

Therapists use a wide range of clinical interventions in their repertoire but there is little research evidence to guide clinical decisions on which patients are likely to respond to which therapies. Possible influential factors include the location and size of brain lesion [28,29], degree of motor weakness; and ability to control the trunk to sit independently [30,31]. It is unknown whether these factors are prognostic for obtaining benefit from pedalling exercise early after stroke.

## Aims

The driver for this proposed research is the hypothesis that UniCam crank-assisted upright pedalling (UP), used as an adjunct to conventional physical therapy, enhances recovery of lower limb motor function in stroke survivors with substantial paresis early after stroke. However, before this hypothesis can be tested in a phase III trial, it is important to establish whether there is sufficient evidence of benefit (clinical efficacy) to justify proceeding to subsequent larger trials and which stroke survivors are most likely to be able to participate in UP (prognostic indicators). Therefore, the aims for the current early phase clinical research study are:

1. Clinical Efficacy

To determine whether there is sufficient evidence for UP, balancing efficacy and potential adverse events (pain and fatigue), to justify proceeding to subsequent larger clinical trials; as assessed by:

a) ability to voluntarily contract paretic muscle;

b) production of reciprocal activation of antagonistic muscle groups during pedalling, similar to walking;

c) timing of onset and offset of activity in antagonist muscle groups during pedalling, similar to walking;

d) ability to walk independently.

2. Prognostic Indicators

To determine whether site of stroke lesion, trunk control ability, severity of lower limb paresis and/or ambulatory ability predicts ability to use UP within 30 days of stroke onset.

## Methods

### Design, setting and randomisation

**Figure 1:**
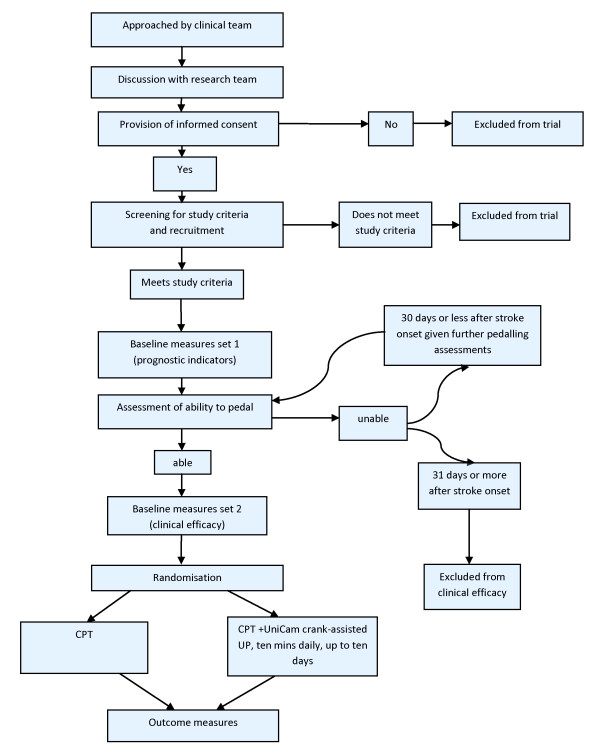
**Flowchart illustrating trial design**.

The proposed study will be a single centre, early phase randomised controlled trial with observer blinding, preceded by an observational component. This design is illustrated in figure 1.

#### Study procedure

All participants will undergo baseline measurement set 1 (prognostic indicators). They will then be assessed for their ability to perform UP. Potential participants will be taken to the treatment area and shown the cycling equipment. If content to proceed, a hoist will be used to seat them on the bike safely. They will be asked to pedal slowly for one minute to familiarise themselves with the equipment. They will then be asked to pedal for one further minute and a visual observation of whether they can pedal or not will be made.

Those unable to pedal and who are 31 days or more after stroke onset will be excluded from the randomised part of this trial. Those unable to pedal and who are 30 days or less after stroke onset will be offered further pedalling assessments approximately every three days. The rationale for further pedalling assessments is that, during the first 30 days after stroke, people may experience fear of movement or emotional distress and therefore may need more than one experience of pedalling within a therapeutic environment. Without repeated opportunities for pedalling assessment some participants may be excluded unfairly from the opportunity to participate in UP.

Those participants able to pedal for one minute and who are 30 days or less after stroke onset, will then undertake baseline measures set 2 (clinical efficacy). Participants will then be allocated randomly to either routine conventional physical therapy (CPT; control group) or to CPT plus UP (experimental group). Randomisation order will be generated before the trial begins by an independent statistician, in blocks of four. Group allocation will be concealed in sequentially numbered opaque sealed envelopes held by an independent administrator, who is not involved in the study and will have no contact with study participants. The next highest number envelope will be opened by the independent administrator in response to a telephone request from the research therapist. After opening, envelopes will be stored securely with the participants' study data. Randomisation will be concealed from the independent outcome assessor and participants will be asked not to discuss group allocation with the outcome assessor.

Participants will receive their allocation intervention for up to ten minutes a day, for up to ten working days or discharge from acute stroke care, whichever occurs first. On completion of the intervention phase, participants will undertake clinical efficacy outcome measures. Every attempt will be made to undertake outcome measures even if participants withdraw or are discharged before the intervention phase is completed (intention to treat principle).

#### Blinding

Blinding of research therapists in a therapy intervention study is not always feasible and patients are clearly aware that they are undergoing therapeutic interventions. Consequently, for this exploration of pedalling exercise, blinding of therapists and participants is not possible. However, the independent assessor of clinical outcome measures will be a trained therapist blinded to group allocation.

### Ethical considerations

Patients with communication deficit (particularly aphasia) are frequently excluded from stroke rehabilitation research, despite having potential for motor benefit. In clinical practice, however, stroke survivors with aphasia are included in motor rehabilitation. This protocol ensures that, providing patients can follow a single-stage command, they can participate. Thus the results of this trial will be applicable to clinical practice. In addition, the protocol addresses a frequent complaint from stroke survivors with aphasia; namely that they are not given opportunities to be involved in research.

However, in clinical practice as well as research, it is important to distinguish between language and cognitive communication impairment and close liaison with the clinical team, in particular the Speech and Language Therapy members, is essential. Before approaching a potential participant, the researcher will therefore discuss decision making capacity of individuals with the clinical team. If, as a result of their assessment, the clinical team's conclusion is that communication impairment is too great to allow an individual to give informed consent, then the researcher will not approach the potential participant. If the clinical team's conclusion is that informed consent is possible, albeit with the use of enhanced communication strategies, then the researcher will approach the potential participant.

Enhanced communication strategies will be used in this trial. These include the use of diagrams, charting information, repetition in a variety of ways and checking for understanding. In addition, information sheets and informed consent forms present information in a textual and pictorial form.

All potential participants will be given at least 24 hours (1 working day) to consider the information and ask questions. They will be encouraged to consult with others, outside of the research team, before making their decision.

All data will be encrypted and then stored on an lap top computer by the researcher before leaving the stroke unit. Data will be transferred onto a secure hard drive in the research laboratory. No names will be used in any recorded material except for the initial screening document. Participants will be anonymised with the use of study ID numbers.

The research study has received the **approval of the Essex 1Research Ethics Committee, UK (09/H0301/52)**.

### Participant inclusion criteria and recruitment process

Participants will be recruited from an acute stroke unit and, if necessary due to pressure on stroke beds, medical wards; in a University Hospital Trust. Consultant and therapy teams have agreed to support this trial.

Stroke survivors will initially be approached by a clinical team member responsible for their care, to check that they agree to speak to a researcher. If they agree, then a researcher will provide potential participants with verbal and written printed information about the trial. A video of the procedure for getting on and off the exercise cycle will also be available if patients wish to view it. A minimum of twenty four hours (1 working day) later, informed, signed consent will be sought. Those providing written informed consent will be participants in this trial. All potential participants will then be screened to check that they meet the study criteria, which are;

• adults aged 18+

• three to thirty days following a unilateral stroke resulting in unilateral muscle weakness with or without sensory deficit;

• fit to participate as assessed by a consultant-led medical team with resting oxygen saturations 95% or above, resting heart rate 90 beats per minute or less and systolic blood pressure of 100-160 mmHg

• score 0, 1 or 2 on the Functional Ambulation Categories [32]. Clinically, this means unable to walk; or need the help of two or more people; or require firm continuous or intermittent support of one person assisting with weight and balance;

• be able to sit unsupported for 30-seconds on the edge of a bed with feet on the floor.

• have sat out of bed in a chair or wheelchair at least once for a continual period of 15-minutes i.e. have appropriate sitting tolerance to participate in this cycling intervention;

• be able to follow a one-stage command i.e. sufficient communication, orientation and memory to participate in this cycling intervention;

• be independently mobile with or without an aid prior to the index stroke;

• have no co-existing pathology contributing to observed impairment in the paretic lower limb e.g osteoarthritis with associated knee deformity.

### Sample Size

This early phase trial is the first to use this equipment and with this participant group. Consequently there are no data to inform a power calculation. Sample size will therefore be based on practical considerations, using estimates of the number of participants we could expect to recruit within a 12 month time period. Using data from our previous trials of rehabilitation earIy after stroke [e.g [33]], we estimate a recruitment rate of two participants per month. Therefore, the sample size has been set at 24 participants.

### Intervention and Instrumentation

All participants will receive routine conventional physical therapy (CPT) as deemed appropriate by the clinical team. To enable replication of CPT we will record its content and dose (minutes of therapy) with a standardised schedule [34].

#### Control intervention

Participants allocated to the control group will receive CPT only as described above.

#### Experimental Intervention

Participants allocated to the experimental group will receive UP in addition to CPT. All experimental participants will be asked to pedal at 50 revolutions per minute (50 rpm) at a comfortable resistance whilst maintaining a heart rate of 85% or below their age-predicted maximum (i.e. less than 220-age ×0.85 beats per minute). If patients cannot achieve 50 rpm, the research therapist will be guided by their response in setting the maximum rpm. The mean rpm achieved will be recorded for each participant for each intervention session. It is anticipated that few patients this early after stroke will immediately manage ten minutes of pedalling, so the number of minutes pedalled, up to ten minutes, will be recorded.

Each intervention session will also involve recording: the pedal crank setting; the degree of reciprocal activation of antagonistic muscle groups (see measurement battery); the timing of onset and offset of activity in antagonistic muscle groups (see measurement battery); and the distance pedalled (m). This description of each intervention session will allow replication of the intervention and information on how to progress the intervention over time in subsequent clinical trials.

#### Instrumentation

**Figure 2:**
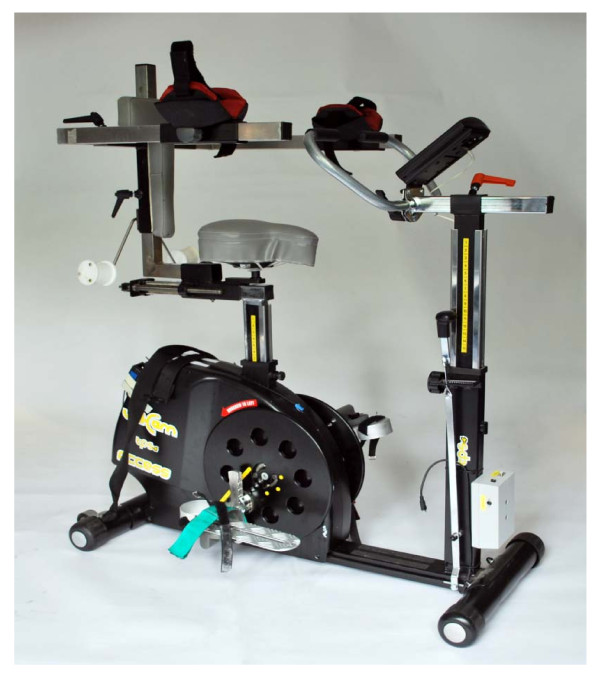
**The modified exercise cycle**.

Maintaining sitting balance early after stroke often requires substantial concentration and physical effort which may limit production of selective movement in the paretic lower limb. We have therefore adapted a standard exercise bike so that postural support for the trunk is provided if needed (figure 2).

We have also incorporated a UniCam crank, an adjustment that can be applied to any commercially available exercise bike and which enables movement of the axis of the crank towards the centre of rotation of the bike pedal. This thereby reduces the circumference of the pedalling circle and reduces the required range of movement at the knee and hip, allowing patients who may have substantial lower limb weakness and/or limitations in the range of joint movement to still pedal.

EMG data will be collected using the *Datalink *system (Biometrics, UK). Muscle activity in quadriceps and hamstring muscles for each leg will be recorded using SX 230 (Biometrics, UK) preamplifiers. The preamplifiers connect to 4 analogue channels of the *Datalink *subject unit, which is connected to the base unit. Information from the base unit is collected on a lap top computer running the *Datalink *software system. Continuous EMG data will thus be recorded during pedalling.

**Figure 3:**
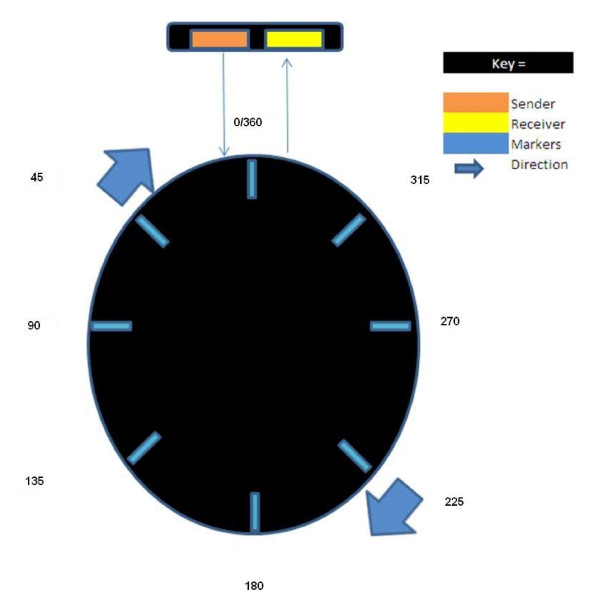
**Diagrammatic representation of wheel demonstrating divisions**.

The bicycle wheel is demarcated every 45 degrees using reflective tape. As the participant pedals, an LED sensor placed at a fixed point on the bicycle frame, is triggered as each of the eight markers passes (figure 3). This trigger creates a drop in voltage, creating a spike in the software. The spikes are recorded synchronously, via a digital channel on the *Datalink *subject unit, with the EMG data. This system allows for muscle activity to be related to the position of the pedal during the 360 degree turn.

### Measurement battery

Baseline measures will be made before randomisation and outcome measures after the intervention phase has been completed (figure 1). Baseline measures consist of two sets: prognostic indicators and clinical efficacy. Outcome measures will consist of clinical efficacy measures only.

The ***participant characteristics ***to be recorded for all potential participants and participants will be: gender, age (years), type and site of the stroke lesion (liaison with medical team from scanning/clinical findings) and time since stroke onset at entry to the trial and at each set of study measures (days).

#### Clinical efficacy measures

As the primary aim of this pedalling intervention is to enhance ability to voluntarily contract paretic muscle, the primary measure enables assessment of impairment level change. The Motricity Index [35] is a simple measure that can be used easily in the clinical setting to assess the severity of motor impairment. It is also a significant predictor of ambulatory outcomes after stroke [30,36]. Hence it is a highly clinically relevant measure, as it provides a direct assessment of motor function that is correlated with eventual mobility outcomes.

To detect changes in muscle activity underlying participants' observed performance, EMG data will be employed. Therapists in the clinical setting frequently observe and record alterations in, for example, muscle strength and walking ability, but cannot accurately measure the biological changes in muscle activity that might contribute to changes in functional performance. In recording, processing and analysing at this level, the proposed trial will be able to evaluate biological change alongside frequently used clinical measures of recovery. This change in motor activity will be able to be detected earlier than if using clinical measures of movement performance alone.

Regaining mobility is a key goal for stroke survivors and independent mobility enables independence in other activities of daily living [9,30]. It is possible that, as pedalling exercise uses similar motor control patterns to those required for walking, UP after stroke might have a positive effect on ambulatory function. A measure of walking ability has therefore been included in this study. The Functional Ambulatory Categories (FAC) [32] has demonstrated sensitivity in stroke survivors who cannot walk at the beginning of their rehabilitation period, applicable to participants in this trial, who are not mobile at inclusion. This measurement of ambulatory function provides an assessment of an activity level change that is highly relevant after stroke and completes a spectrum of measures for this trial from body structure through function to activity.

##### Primary outcome

1. Ability to voluntarily contract paretic muscle

This will be measured by the Motricity Index (MI) lower limb section [35]. The MI is a widely used measure and has established validity and reliability for use after stroke [37]. It is an ordinal weighted scale with six measurement levels within each of three categories for the lower limb. The three categories are: ankle dorsiflexion, knee extension, and hip flexion. For each movement, a score of 0, 9, 14, 19, 25, or 33 is given, where 0 is no movement, 19 is full range movement against gravity not against resistance and 33 is normal power.

##### Secondary outcomes

2. Ability to walk independently

As measured by the Functional Ambulation Categories (FAC) [32]. This scale is designed to give detail on physical support needed by patients for walking, so has clinical relevance, and is easy to use. It has established validity and reliability for use after stroke [38]. It is an ordinal scale, patients scoring from 0-5, where 0 indicates a patient who is not able to walk or needs help of 2 therapists, and 5 indicates a patient who is independent in ambulation even on stairs.

3. Onset and offset of EMG activity of antagonistic muscle groups during pedalling

EMG activity will be recorded in quadriceps and hamstring muscles for each leg. Before getting on the bike, participants will have a small (37 mm × 18 mm) pre-amplifier applied to the front and back of their thigh on both sides, following skin preparation to minimise signal interference. Electrode position is known to be a vital factor in achieving accurate EMG information [39]. For this study, a single researcher will place the electrodes for each participant and for every session, using published guidelines [40]. When the participants are positioned comfortably on the bike, the leads from the pre-amplifiers will be connected as described in *instrumentation*.

Resting EMG activity will be recorded as a voltage at 1,000 Hz whilst the participant's foot is resting firmly on a box so that the leg is still and supported with the knee in 5-15 degrees of flexion, for 30 seconds. This will be undertaken for each leg. EMG data (voltage) will be collected continuously during pedalling for a minimum of 30 seconds at approximately 50 rpm.

Baseline EMG values will be calculated from the rectified, processed signal as the mean ± 3 SD (standard deviations) during the 30 seconds baseline data collection period. Onset of activity in each of the four muscle groups will be defined as the time point during the 360 degree turn at which EMG voltage exceeds the mean baseline value plus 3SD for 20 consecutive data points (20 ms). Offset of activity in each of the four muscle groups will be defined as the time point during the 360 degree turn at which EMG voltage falls below the mean baseline value minus 3SD for 20 consecutive data points (20 ms). The time point for onset and offset of muscle activity in each of the four muscle groups will also be recorded as a function of the position of the pedal during the 360 degree turn.

4. Reciprocal activation of antagonistic muscle groups (muscle activity) during pedalling

Rectified EMG data for each antagonistic muscle group will be analysed using Spearman's correlation coefficient. An r value of 1.0 indicates perfect positive correlation and therefore complete co-contraction, no reciprocal activation, of an antagonistic muscle pair. An r value of 0 indicates no correlation and therefore no relationship between EMG activity of an antagonistic muscle pair. An r value of -1.0 indicates a perfect negative correlation and therefore complete reciprocal activation of antagonistic muscle groups.

#### Prognostic indicator measures

5. Site of stroke lesion

The location and size of stroke lesion have been demonstrated to be a prognostic factor for functional outcomes after stroke [28,29]. It is possible, therefore, that this clinical factor might be linked to the ability to take part in rehabilitation interventions. Brain lesion location will therefore be recorded from the clinical scan.

6. Degree of muscle weakness as measured by the Motricity Index (see clinical efficacy measures)

7. Ambulatory Capacity as measured by the Functional Ambulatory Categories (see clinical efficacy measures)

The FAC has been found to have good predictive validity for community ambulation after stroke (FAC ≥ 4 predicts community ambulation at six months with 100% sensitivity and 78% specificity) [38]. It is proposed that pedalling exercise might have a positive effect on walking and thus postulated that the ability to walk might influence the ability to pedal and respond to pedalling intervention.

8. Ability to control the trunk

As measured by the Trunk Control Test [37]. This is a short, simple measure of motor loss developed for use after stroke. Patients are asked to do four movements-rolling to their weak side, rolling to their strong side, sitting up from lying down and balancing in a sitting position. Each movement is scored according to ability, either 0, 12 or 25, leading to a total score out of 100. Validity and reliability (comparison with Rivermead Motor Assessment at six, twelve and eighteen weeks post-stroke-Spearman's rho, *r*= 0.70, 0.72 and 0.79 respectively; interrater reliability, Spearman's rho, *r = *0.76, p < 0.001) have been established [37].

Balance (trunk) control is highly specific to ambulatory control, and makes a crucial contribution to the ability to perform activities of daily living [41]. The Trunk Control Test has been found to be a predictor of functional outcomes after stroke, including significant correlation with: discharge Functional Independence Measure (Pearson's *r *= 0.738) and gait velocity (Pearson's *r *= 0.654) [31]; and discharge walking ability (Spearman's rho = 0.71) [36]. It is possible, therefore, that trunk control early after stroke might influence the ability to perform rehabilitation activities and thus will be assessed as a potential prognostic indicator for pedalling exercise after stroke.

#### Adverse events

There is a small risk that for some people, UP might lead to an "overuse" syndrome, as expressed through an increase in pain or fatigue. We will monitor for this by checking for participant reports of lower limb pain, either verbal or behavioural. Intervention will cease and an adverse event recorded if a participant demonstrates a decrease of 2 or more minutes in ability to pedal on 2 consecutive treatment days, or a 25% reduction in mean rpm on 2 consecutive treatment days.

## Statistical Analysis

The aim of the analysis is not to definitively demonstrate efficacy in this early phase trial. Rather the data will be used to inform a decision on whether or not to undertake subsequent studies of UP. Assuming a normal distribution, independent t-tests will be used to compare groups between trial arms for follow-up measures, together with 95% confidence intervals to inform preliminary conclusions on clinical benefit. Within-group analysis will be assessed using paired t-tests. If a normal distribution cannot be assumed, analogous non-parametric methods will be used.

Associations between potential prognostic indicators and the ability to pedal will be examined using Fishers Exact test.

## Trial management

A Trial Management Group (TMG) will provide overall supervision and ensure good conduct of the trial (i.e. adherence to the Declaration of Helsinki). The TMG will meet every three months during the course of this trial. In accordance with the MRC code of good practice in clinical trials and the CONSORT guidelines, we will document all decisions regarding eligibility for entry, consent giving, inclusion, exclusion and attrition. Members of the TMG will be: the researcher (NH) and members of the research team (VP, LS, PR, PKM). Every six months during data collection, the TMG will include an invited independent patients' advocate from the clinical stroke service.

## Discussion

This protocol describes an original, two-stage early phase trial, in which a group of early stroke survivors will first be evaluated for their ability to pedal a modified upright exercise cycle. Those who can pedal the cycle will then be participants in an early phase randomised controlled trial of daily pedalling intervention, for up to ten subsequent working days of their in-patient hospital stay.

Findings from neuroimaging studies suggest that rehabilitation programs incorporate repetition, motor skill acquisition and functional activity in order to optimally drive useful cortical plasticity [e.g. [10-14]]. It has been suggested that early rehabilitation intervention might exploit a crucial period in which the brain is primed to begin repair, in the first few days after stroke onset [1,2]. Therapists are therefore challenged to find rehabilitation strategies incorporating these underlying principles. Cycling provides a paradigm through which such activity might be achieved even in early stroke participants with severe weakness. For this trial, a prototype upright exercise cycle has been developed to enable such patients to experience bilateral pedalling motion. The locomotor strategies employed during cycling are akin to those used in ambulation [18] and our exercise cycle also incorporates adaptations to allow stroke survivors with considerable weakness to attempt to pedal in an upright posture, similar to walking.

Whilst evidence exists correlating clinical aspects of stroke to functional outcomes [e.g. [28-30]], prognostic information on what factors might influence the ability to take part in specific rehabilitation activities has yet to be established. This information has the potential to inform the design of future research and provide indicators to clinicians about which patients might best take part in which activities. The current trial will record four potential prognostic indicators-site of lesion, trunk control, paretic leg motor function and walking ability-before participants attempt to use the equipment; links to the ability to pedal the pedalling activity will be analysed and contribute to clinical conclusions and inform future research. For this novel aspect of the study, selection of potential indicators was based on those factors previously demonstrated to correlate to functional outcomes after stroke.

Some exploratory studies have investigated the potential clinical efficacy of pedalling exercise after stroke [20,24,25] but the early findings of our systematic review (in progress) suggest that no trial has evaluated upright pedalling in a group of stroke survivors within one month of stroke onset. The potential challenges that early stroke survivors might face in taking part in this activity, such as safely sitting in an upright posture and taking part in repetitive exercise, have been addressed: firstly by using a modified exercise cycle, and secondly, by ensuring that physiological parameters and evidence of fatigue are monitored and recorded by the research team.

It is possible that our results might indicate none of the prognostic indicators are linked to the ability to pedal, and/or clinical efficacy of the intervention is not demonstrated. If this is the case, the risk of wasting valuable research resources on larger-scale trials, using the current indicators and measures, is minimised. However, interpretation of findings, whether negative or positive, will reflect the small sample size and early phase nature of this work.

A further novel aspect is that this study of pedalling exercise incorporates biological level measures, alongside more frequently used clinical, functional measures. EMG data from quadriceps and hamstrings will be recorded at baseline and outcome as well as at each pedalling session, providing evidence of any change at a biological level that might contribute to, and underpin, possible changes in functional measures. Using sessional EMG recordings will also allow analysis of whether pedalling is being achieved by the unaffected leg propelling the crank i.e with the use of compensatory strategies, or whether there are changes in recordable activity in the affected leg suggestive of recovery.

The control group will undergo conventional therapy only, and this will be quantified on a standardised treatment schedule, allowing for comparisons of amount and type of therapy across trial arms. Concern has been expressed that reporting of research into complex interventions often lacks sufficient detail on comparators [42]. The use of careful recording of conventional therapy in this trial will go some way towards addressing these concerns and might provide important information for potential dose-matching in later phase work.

This trial is being carried out in an acute stroke unit, and uses portable EMG equipment so that all trial measures can be taken on site. This enables participants to take part in an active rehabilitation setting and hence exploration of the feasibility of the use of the modified bicycle in a busy therapeutic environment; and ensures close collaboration between clinical and research teams for the duration of data collection.

In summary, the proposed novel, early phase research will increase knowledge of prognostic indicators for, and clinical efficacy of, upright pedalling exercise early after stroke. It will provide essential information for the design of subsequent trials.

## Abbreviations

UP: UniCam crank-assisted upright pedalling; RCT: Randomised Controlled Trial; CPT: Conventional Physical Therapy; FAC: Functional Ambulatory Categories; MI: Motricity Index; TMG: Trial Management Group

## Competing interests

The authors declare that they have no competing interests.

## Authors' contributions

NH, LS, and VP participated in the study design. PKM provided medical clinical collaboration at the site and contributed to study design. PR provided bioengineering design support. NH drafted the manuscript and is the lead researcher at the site. VP and PKM contributed to the final draft of the manuscript, and it was approved by all authors.

NH is undertaking this work as part of a PhD at the University of East Anglia, UK.
